# CSF and serum inflammatory response and association with outcomes in spontaneous intracerebral hemorrhage with intraventricular extension: an analysis of the CLEAR-III Trial

**DOI:** 10.1186/s12974-021-02224-w

**Published:** 2021-08-21

**Authors:** Aaron M. Gusdon, Carol B. Thompson, Kendel Quirk, Yunis M. Mayasi, Radhika Avadhani, Issam A. Awad, Daniel F. Hanley, Wendy C. Ziai

**Affiliations:** 1 Division of Neurocritical Care, Department of Anesthesiology and Critical Care Medicine, Johns Hopkins University School of Medicine, 600 N. Wolfe St./Phipps 455, Baltimore, MD 21287 USA; 2 Present address: Department of Neurosurgery, University of Texas Health Science Center at Houston, Houston, TX USA; 3 Johns Hopkins Biostatistics Center, Bloomberg School of Public Health, Baltimore, USA; 4 Division of Brain Injury Outcomes (BIOS), Johns Hopkins University School of Medicine, Baltimore, USA; 5 Department of Neurosurgery, University of Chicago Pritzker School of Medicine, Chicago, IL USA

**Keywords:** Intracerebral hemorrhage, Intraventricular hemorrhage, Inflammation, Fibrinolysis, Clinical outcomes

## Abstract

**Background:**

Intracerebral hemorrhage (ICH) results in a cascade of inflammatory cell activation with recruitment of peripheral leukocytes to the brain parenchyma and surrounding the hematoma. We hypothesized that in patients with ICH and intraventricular hemorrhage (IVH), a robust cerebrospinal fluid (CSF) inflammatory response occurs with leukocyte subtypes being affected by alteplase treatment and contributing to outcomes.

**Methods:**

Serum and CSF cell counts from patients in the phase 3 Clot Lysis: Evaluating Accelerated Resolution of Intraventricular Hemorrhage (CLEAR III) trial were analyzed. CSF leukocytes were corrected for the presence of red blood cells. Trends in cell counts were plotted chronologically. Associations were evaluated between serum and CSF leukocyte subtypes and adjudicated functional outcome (modified Rankin Scale; mRS) at 30 and 180 days and bacterial infection according to treatment with intraventricular alteplase versus saline.

**Results:**

A total of 279 and 292 patients had ≥3 differential cell counts from serum and CSF, respectively. CSF leukocyte subtypes evolved during IVH resolution with a significantly augmented inflammatory response for all subtypes in alteplase- compared to saline-treated patients. CSF leukocyte subtypes were not associated with detrimental effect on functional outcomes in the full cohort, but all were associated with poor 30-day outcome in saline-treated patients with IVH volume ≥20 mL. Higher serum lymphocytes were associated with good functional outcomes (mRS 0–3) in the entire cohort and saline-treated but not alteplase-treated group. Conversely, increased serum neutrophil-to-lymphocyte ratio (NLR) in the entire cohort and saline group was associated with worse functional outcomes. Higher median serum lymphocytes were associated with the absence of infection at 7 days.

**Conclusions:**

Aseptic CSF inflammation after IVH involves all leukocyte subtypes. Serum lymphocytes may be associated with better outcomes by mitigating infection. Alteplase augments the inflammatory response without affecting outcomes.

**Supplementary Information:**

The online version contains supplementary material available at 10.1186/s12974-021-02224-w.

## Background

Intracerebral hemorrhage (ICH) results in high rates of morbidity and mortality [1, 2], with intraventricular hemorrhage (IVH) being associated with poor functional outcomes [3, 4]. ICH initiates a cascade of inflammatory cell activation, with recruitment of leukocytes to the brain parenchyma surrounding the hematoma [5]. A sterile cerebrospinal fluid (CSF) inflammatory response associated with IVH lasting 3–5 days has been previously reported by this group and others [6–8]. Treatment of IVH with intraventricular thrombolysis has led to further investigation of the CSF inflammatory response with several studies finding a significantly augmented response in the presence of intraventricular alteplase [7, 9, 10].

We previously demonstrated increased CSF white blood cell (WBC) counts after intraventricular alteplase compared to saline administration in subjects enrolled in the phase 3 Clot Lysis: Evaluating Accelerated Resolution of Intraventricular Hemorrhage (CLEAR III) trial, but without an effect on clinical outcomes [8]. Leukocyte subtypes were not analyzed in that study.

Serum leukocyte populations have different relationships with hematoma expansion and functional outcomes, with monocytes associated with increased mortality and hematoma expansion, and neutrophil-to-lymphocyte ratio (NLR) associated with higher 30-day mortality, poor 3-month functional outcomes, and early neurologic deterioration [11–14]. The contribution of CSF leukocyte populations to functional outcomes after IVH and their association with intraventricular alteplase has not been investigated.

We hypothesized that a robust CSF inflammatory response would occur after intraventricular alteplase administration affecting all subtypes and that elevated NLR would be associated with worse outcomes with lymphocytes playing a protective role.

## Methods

### Study design and eligibility criteria

This is a prospective observational cohort study of collected CSF from patients enrolled in the CLEAR III trial (ClinicalTrials.gov, NCT00784134) [15]. The study protocol was approved by the appropriate institutional review board at each participating site, and written informed consent was obtained from all patients (or legal representatives or surrogates when applicable).

All patients enrolled in CLEAR III had ICH volume less than 30 mL with IVH causing obstruction of the third and/or fourth ventricle. Patients with underlying vascular etiology were excluded. All patients were treated with a standard of care external ventricular drain (EVD) and were randomized to treatment with intraventricular saline or alteplase (1 mg) three times daily (every 8 h) for up to 12 doses, with treatment stopped after achieving clearance of the third or fourth ventricle or 80% or more of baseline IVH volume [15]. Enrolled patients had protocolized daily CSF samples collected from the EVD with analysis for cell count (cells/μL), protein, and glucose (mg/μL). Gram staining and cultures were added if bacterial infection was suspected. Absolute counts of total WBCs, neutrophils, monocytes, and lymphocytes were obtained from differentials obtained from clinical laboratory testing at each trial site. Differential cell counts were not mandated, with the decision to measure serum/CSF differentials being made by the clinical team. Data were abstracted from sequential laboratory data collected by each clinical site at randomization and for the next 5 days. Cell counts including WBC, neutrophils, monocytes, and lymphocytes as well as red blood cells (RBCs) were collected from both the serum and CSF. For this study, we included patients who had ≥3 daily values for each leukocyte subtype. No imputation of unavailable data was performed.

#### CSF cell count correction

CSF WBC counts were corrected as previously described by subtracting predicted from observed CSF WBC counts.^18^ Predicted CSF WBC counts were calculated as follows: CSF WBC_predicted_ = CSF RBC count × [(peripheral WBC count)/(peripheral RBC count)]. Each leukocyte value was corrected in the same fashion. Negative values after correction were assigned a value of 0.

#### Outlier determination

Outliers for a given leukocyte were defined as values greater than 2.5 standard deviations above the daily mean across all available samples. Potential outliers were evaluated against the patient’s values on days immediately preceding and following that value. Values considered to be outliers (52 for CSF WBCs, 35 for CSF neutrophils, 29 for CSF monocytes, and 31 for CSF lymphocytes) were deemed erroneous readings and were excluded from analysis.

#### Radiographic and outcome variables

IVH volumes were measured from protocolized computed tomography (CT) scans at the CLEAR III’s core image reading center using semiautomated segmentation and Hounsfield Thresholds [15]. Initial IVH volume was quantified from the last CT scan prior to randomization (stability scan), and end of treatment IVH volume from the CT scan obtained 24 h after the last administration of either saline or alteplase. Percent IVH clearance was quantified as 100* (initial IVH volume – end of treatment IVH)/initial IVH volume. Functional outcomes were assessed using the modified Rankin Scale (mRS) at 30 and 180 days after symptom onset with independent adjudication at the trial’s outcomes center, University of Glasgow, Scotland, UK. Dichotomization of mRS scores was defined as good (mRS 0–3) and poor (mRS 4–6) outcomes, as prespecified in the trial. Infections (urine, respiratory, bloodstream, and CSF) occurring within the first 7 days from randomization were strictly defined and reported [15]. Bacterial CSF infection (ventriculitis) was defined as a positive culture from CSF within the first 7 days following randomization. All other demographic and baseline variables were recorded at randomization.

#### Statistical analysis

Descriptive statistics (medians and interquartile ranges [IQR] or percentages) were calculated to compare the population with ≥3 CSF cell count differentials by treatment group and compared with the full CLEAR III population. Comparisons were also made between patients with ≥3 and <3 CSF differential cell counts available. Categorical variables were compared using Pearson χ^2^ test, while continuous variables were compared using a two-sided *t* test or Mann-Whitney test.

The large number of zero values in CSF cell counts after correction produced a skewed, heteroscedastic distribution. Zero-inflated values yield non-parametric distributions, with significant effects on models. Methods for dealing with zero-inflated distributions are complex [16, 17]. Therefore, we decided to exclude zero values from analysis. For trend data, models were analyzed (1) including all data to compare the number of zero and non-zero values at each time point and (2) excluding zero values. For outcomes, models were also analyzed with these two approaches.

Daily cell counts were analyzed using general linear regression models of daily counts and treatment, accounting for within-patient correlation of daily counts. One type of analysis considered days as a factor with day-by-treatment interactions to evaluate treatment differences in counts at each day. A second analysis was performed with day as continuous to compare the slopes of cell counts across days between the treatments.

Leukocyte cell counts were calculated as the median from the day of randomization through the first 5 days post-randomization, for patients that had ≥3 daily values. Logistic regression models evaluated the relationship between each serum or CSF leukocyte’s median cell count and dichotomized mRS outcomes at 30 and 180 days. All analyses were adjusted for clinical severity using the severity index developed for the CLEAR III trial, which includes categories for well-established explanatory variables: age, Glasgow Coma Scale (GCS) at randomization, ICH location (thalamic versus other), and ICH and IVH volume [15]. Logistic regression analyses were adjusted for percent IVH clearance. Outputs from linear regression models are reported as β (slope) coefficients with 95% confidence intervals (95% CI); logistic regression models are reported as odds ratios (OR) with 95% CIs.

Sensitivity analysis evaluated associations of leukocyte subtypes with functional outcomes in patients with initial IVH volume ≥20 mL, a pre-specified more severe population, which benefited to a greater extent from thrombolytic treatment in the CLEAR III trial [15, 18]. Associations between median and maximum lymphocyte counts and occurrence of 7-day infections were analyzed using the Mann-Whitney test. Statistical analyses were performed using STATA version 15.1 (STATA Corp, College Station, TX, 2019). *P* values are 2-sided and considered statistically significant if *P*<0.05.

## Results

### Patient selection and demographics

Among the 500 patients in the CLEAR III trial, 464 and 400 patients had serum and CSF WBC counts available, respectively. Among patients with WBC data, 279 and 292 patients had ≥3 daily serum and CSF differential cell counts, respectively (Supplementary Table I). Patients with ≥3 days CSF differential cell counts compared to the total CLEAR III population, and patients without any differential cell counts (*N*=101) compared to those with any differential cell counts available (*N*=363) showed no differences in demographic and baseline variables (Table 1). Patients with ≥3 days of differentials available had higher median GCS (9.7 [IQR 9.3–10.1] versus 8.9 [IQR 8.4–9.5], *P*=0.027) compared to patients with <3 days of differentials (Supplementary Table II). Forty-three patients were diagnosed with bacterial ventriculitis within 30 days of randomization. Eighteen of these patients had ventriculitis within the first week after randomization and were excluded from all analysis.

**Table 1:** Patient demographics, comparing CLEAR III cohort with cohort having at least 3 CSF differential cell counts available

	Total CLEAR III Cohort		Cohort with ≥3 CSF differentials	
	Alteplase (***n***=249)	Saline (***n***=251)	Alteplase (***n***=139)	Saline (***n***=153)
Age (years)	59 (51–66)	59 (51–67)	57 (46–63)	58 (51–66)
Women	105 (42%)	117 (47%)	63 (45%)	71 (46%)
Race				
White	144 (58%)	161 (64%)	74 (53%)	87 (57%)
Black	92 (37%)	78 (31%)	59 (42%)	58 (38%)
Native American	0	1 (<1%)	0	0
Other	13 (5%)	11 (4%)	6 (4%)	8 (5%)
Ethnicity: Hispanic	28 (11%)	32 (13%)	15 (11%)	17 (11%)
Baseline variables				
Tobacco use	73 (29%)	59 (24%)	40 (29%)	41(27%)
Cocaine	12 (5%)	18 (7%)	7 (5%)	12 (8%)
Anticoagulation	20 (8%)	29 (12%)	15 (11%)	20 (13%)
Hypertension	168 (67%)	202 (80%)	90 (65%)	122 (80%)
Hyperlipidemia	240 (96%)	245 (98%)	132 (95%)	151 (99%)
Antiplatelet use	56 (22%)	72 (29%)	28 (20%)	38 (25%)
Mean arterial pressure	96 (86–106)	94 (86–104)	96 (88–108)	95 (87–105)
Glasgow Coma Scale	10 (7–13)	9 (7–12)	10 (7–13)	10 (7–13)
NIH Stroke Scale	19 (11–32)	20 (11–35)	18 (9–32)	19 (10–33)
Stability CT				
IVH volume (mL)	21.2 (12.7–34.2)	22.4 (12.7–39.1)	21.3 (13.7–35.3)	24.8 (16.4–39.6)
ICH volume (mL)	8.3 (2.9–15.2)	7.2 (2.3–14.7)	8.3 (2.8–15.2)	7.7 (2.0–14.2)
Index clot location				
Thalamus	149 (60%)	144 (57%)	75 (54%)	83 (54%)
Primary IVH	19 (8%)	27 (11%)	17 (12%)	16 (10%)
Time, hours since ICH				
Hospital arrival	1.5 (0.8–3.3)	1.5 (0.8–3.6)	1.5 (0.8–3.3)	1.6 (0.8–3.7)
First CT	2.3 (1.3–4.6)	2.3 (1.4–5.2)	2.3 (1.3–4.7)	2.4 (1.4–5.6)
First EVD	7.1 (4.5–11.9)	7.9 (5.1–12.0)	6.8 (4.5–10.6)	7.8 (5.1–11.0)
Stability CT	43.1 (25.4–58.8)	44.0 (28.2–57.0)	43.6 (23.9–58.6)	43.8 (28.1–55.5)
Randomization	51.8 (36.4–65.8)	52.2 (41.2–66.8)	52.0 (35.9–66.3)	52.0 (40.9–66.8)

Data are median (interquartile range) or numbers (%). CSF indicates cerebrospinal fluid; *CT*, computed tomography; *EVD*, external ventricular drain; *ICH*, intracerebral hemorrhage; and *IVH*, intraventricular hemorrhage

### Cell count trends

**Fig. 1:**
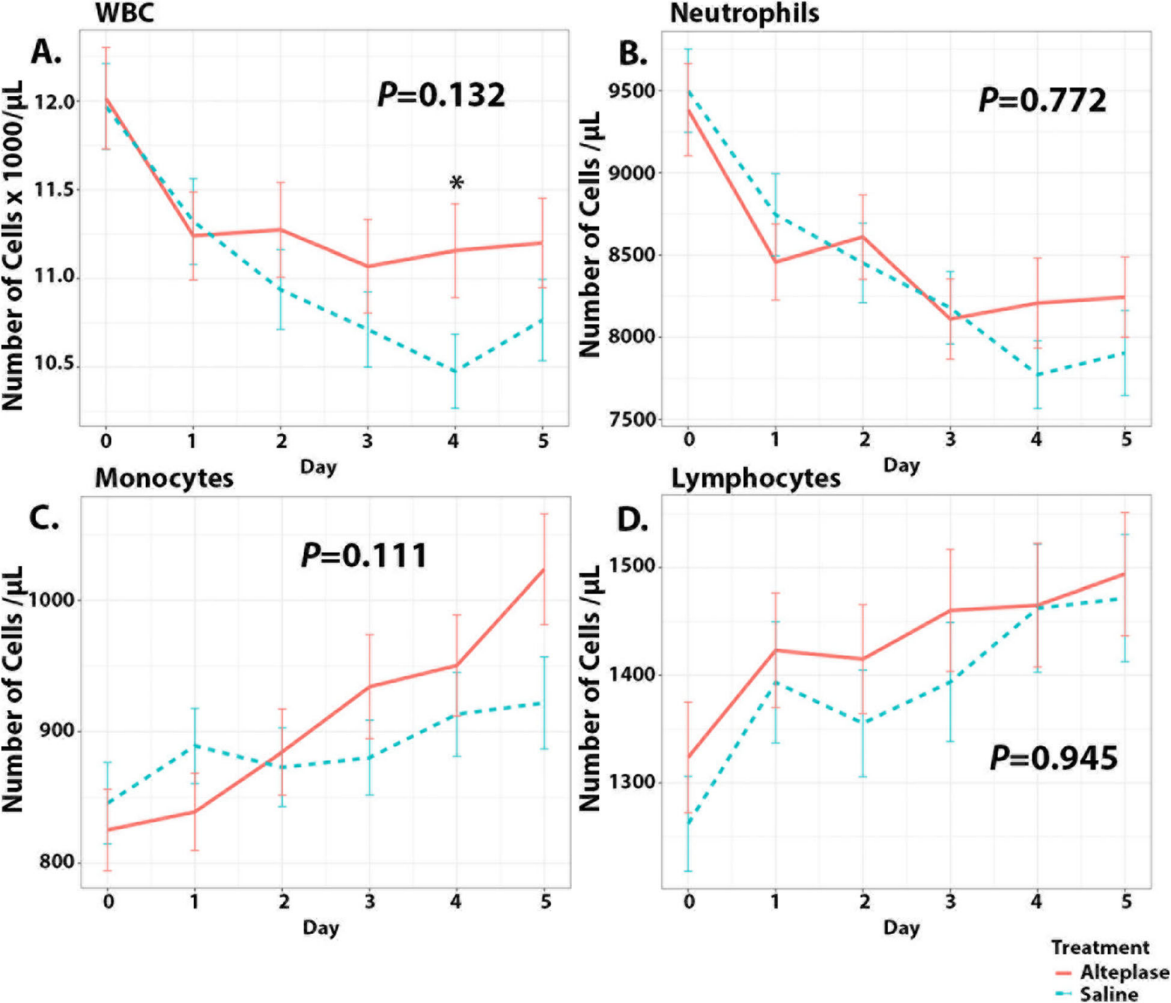
Trends in serum leukocyte counts. Mean cell counts with standard deviations are shown for both alteplase (red) and saline (blue) groups. Day 0 represents the day of randomization and first day of treatment with subsequent values over the next 5 days. *P* values are shown for each cell type and represent differences in overall trends (slopes) between the two groups. Statistical significance: *P<0.05 comparing alteplase and saline treated groups

**Fig. 2:**
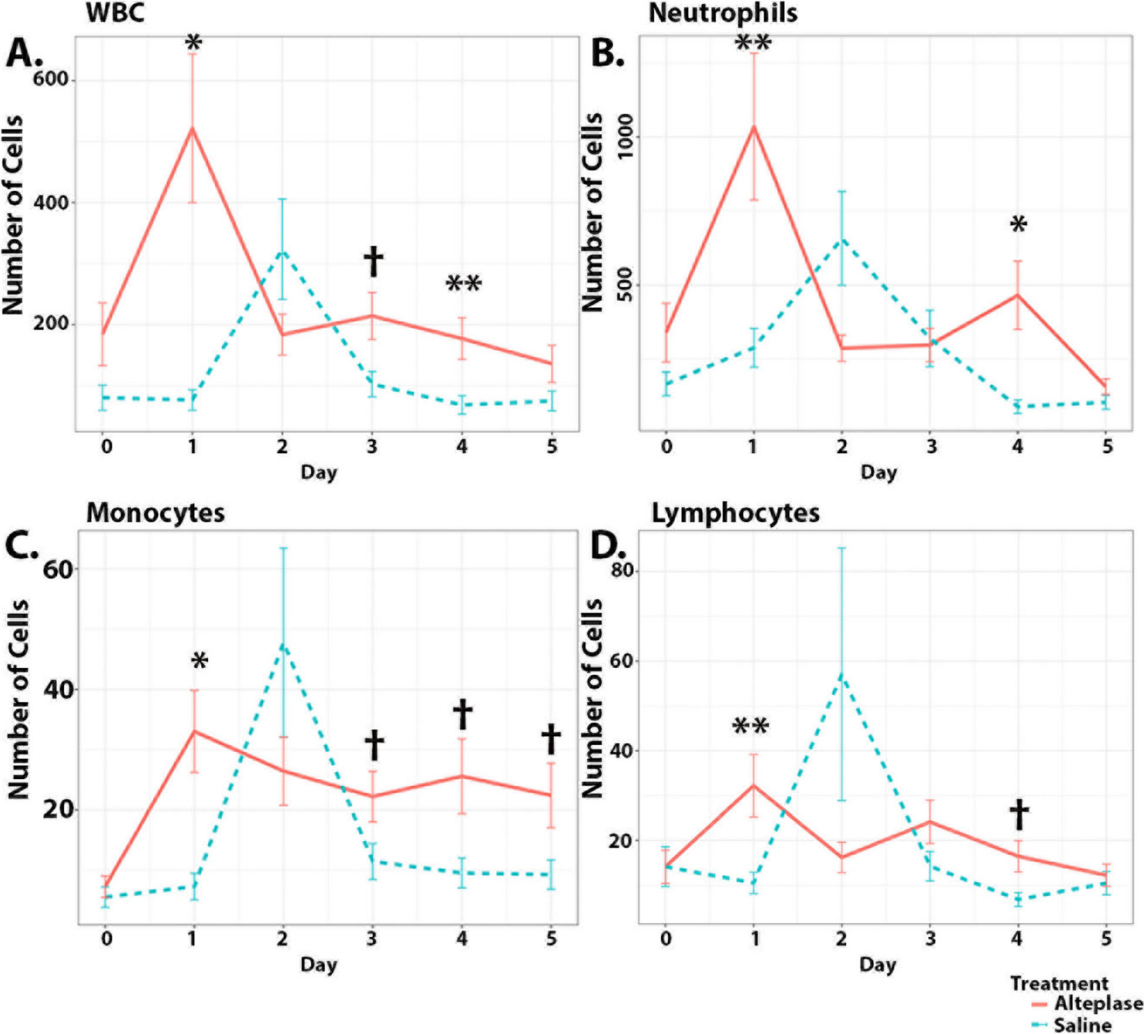
Differences in CSF leukocyte counts by day. Mean cell counts with standard deviations are shown for both alteplase (red) and saline (blue) groups. Day 0 represents the day of randomization and first day of treatment with subsequent values over the next 5 days. Statistical significance: *P≤0.001, **P<0.005, †*P<*0.05, comparing alteplase versus saline-treated groups

Trends in serum cell counts and corrected CSF cell counts are shown in Figs. 1 and 2. There were significant negative trends over time for serum WBC count for both saline (*β=*−0.256 [95% CI, −0.357, −0.155], *P*<0.001) and alteplase groups (*β*=−0.131 [95% CI, −0.223, −0.040], *P*=0.005) (Fig. 1A). There was no difference in the overall slopes between the two groups (*P=*0.132). Considering day-to-day values, serum WBC count was significantly higher in the alteplase group than in the saline group only on day 4 (difference = 0.679 [95% CI, 0.020–1.34], *P=*0.044). Serum neutrophil count demonstrated a significant negative slope in both saline (*β*=−323.805 [95% CI, −436.188, −211.422], *P*<0.001) and alteplase groups (*β*=−201.101 [95% CI, −298.819, −103.384], *P*<0.001) (Fig. 1B) with no difference in the slopes between groups (*P=*0.772). Monocyte counts increased over time with significant positive slope in the alteplase group only (*β*=39.166 [95% CI, 23.832–54.501], *P*<0.001) (Fig. 1C). There was no overall difference in the trends of cell counts between the two groups (*P=*0.111). There was a significant positive trend in serum lymphocyte count in both saline (*β*=37.295 [95% CI, 15.728–58.863], *P*=0.001) and alteplase groups (*β*=29.372 [95% CI, 10.736–48.007], *P*=0.002) (Fig. 1D) with no overall difference in trends (*P=*0.945).

Trends in corrected CSF cell counts are shown in Fig. 2. There were significant differences between saline and alteplase groups in the overall trends in total and all leukocyte subtypes in CSF (Fig. 2A, D). Day-to-day differences are summarized in Supplementary Table III, and some significant day-to-day differences were identified. CSF WBC count was significantly higher in the alteplase group at days 1, 3, and 4. CSF neutrophil count was significantly higher in the alteplase group at days 1 and 4. CSF monocyte count was significantly higher in the alteplase group at days 1, 3, 4, and 5. CSF lymphocyte count was significantly higher in the alteplase group at days 1 and 4. Trends in CSF cell counts including only those values greater than zero are shown in Supplementary Fig. 1. Analysis was also performed including only non-zero values (Supplementary Table IV). The proportion of non-zero values at each day is shown in Supplementary Table V. The odds ratio of a value being non-zero comparing the alteplase with the saline group increased with days after randomization as the alteplase group tended to have more non-zero values later after randomization (Supplementary Table V).

### Associations with outcomes

No associations between mRS and serum total WBC, neutrophil, or monocyte counts at days 30 or 180 were noted (Table 2). Higher serum lymphocyte count was associated with lower odds of poor functional outcome at 180 days in all patients (*P=*0.047) and in the saline group (*P*=0.002). Higher serum NLR was associated with poor functional outcomes at 180 days in all patients (*P=*0.042) and in the saline group (*P*=0.003). These associations were not seen in the alteplase group. We found no significant associations between mRS and CSF leukocyte subtype counts. Scatter plots of cell counts for lymphocytes and NLR in association with 180-day outcomes are shown in Supplementary Fig. 2 (A–F).

**Table 2:** Odds ratios (95% confidenceintervals) for leukocyte subtypes and poor functional outcome (mRS 4–6)

		All patients			Saline			Alteplase		
**30 days**	**Cell type**^**#**^	***N***	OR (95% CI)*	***P***	***N***^†^	**OR (95% CI)**	***P***	***N***	**OR (95% CI)**	***P***
**Serum**	WBC	375	1.03 (0.93–1.13)	0.620	184	1.08 (0.93–1.25)	0.292	191	0.98 (0.87–1.10)	0.727
	Neutrophils‡	245	1.01 (0.99–1.02)	0.549	129	1.00 (0.98–1.03)	0.787	116	1.01 (0.98–1.03)	0.574
	Monocytes‡	248	0.95 (0.83–1.08)	0.420	129	0.93 (0.76–1.15)	0.509	119	0.97 (0.82–1.15)	0.697
	Lymphocytes‡	248	0.97 (0.91–1.04)	0.443	129	1.00 (0.91–1.10)	0.983	119	0.95 (0.86–1.05)	0.282
	NLR	245	1.09 (0.94–1.25)	0.259	129	1.03 (0.85–1.24)	0.767	116	1.17 (0.92–1.49)	0.212
**CSF**	WBC‡	140	1.12 (0.81–1.54)	0.478	62	1.80 (0.56–5.73)	0.323	78	1.07 (0.78–1.47)	0.673
	Neutrophils‡	131	1.10 (0.77–1.56)	0.610	55	1.57 (0.57–4.32)	0.380	76	1.05 (0.79–1.39)	0.729
	Monocytes	118	1.01 (0.98–1.04)	0.512	52	1.08 (0.93–1.26)	0.304	66	1.01 (0.98–1.03)	0.689
	Lymphocytes	116	1.19 (0.28–5.19)	0.812	48	1.75 (0.99–2.80)	0.081	68	1.04 (0.99–1.10)	0.108
	NLR	86	0.96 (0.88–1.05)	0.352	36	0.88 (0.73–1.05)	0.142	50	0.98 (0.88–1.10)	0.748
**180 days**	**Cell type**	***N***	**OR (95% CI)**	***P***	***N***	**OR (95% CI)**	***P***	***N***	**OR (95% CI)**	***P***
**Serum**	WBC	317	1.01 (0.91–1.11)	0.872	148	1.05 (0.90–1.22)	0.510	169	0.98 (0.88–1.10)	0.775
	Neutrophils‡	208	1.01 (0.99–1.02)	0.321	106	1.00 (0.98–1.02)	0.915	102	1.02 (0.99–1.04)	0.254
	Monocytes‡	209	0.96 (0.85–1.07)	0.452	106	0.97 (0.82–1.14)	0.693	103	0.96 (0.82–1.12)	0.575
	Lymphocytes‡	210	0.94 (0.89–0.99)	**0.047**	106	0.87 (0.80–0.95)	**0.002**	104	1.06 (0.97–1.16)	0.233
	NLR	208	1.13 (1.00–1.28)	**0.042**	106	1.31 (1.10–1.56)	**0.003**	102	1.00 (0.83–1.19)	0.966
**CSF**	WBC‡	114	1.04 (0.97–1.10)	0.275	47	0.92 (0.63–1.33)	0.652	67	1.05 (0.96–1.15)	0.303
	Neutrophils‡	108	1.16 (0.96–1.40)	0.113	42	1.19 (0.54–2.63)	0.669	66	1.15 (0.96–1.37)	0.123
	Monocytes	96	1.00 (0.98–1.02)	0.737	40	0.99 (0.97–1.02)	0.663	56	1.00 (0.98–1.03)	0.775
	Lymphocytes	94	1.01 (0.98–1.03)	0.483	36	1.01 (0.96–1.06)	0.600	58	1.01 (0.98–1.03)	0.550
	NLR	71	0.99 (0.93–1.07)	0.880	28	0.98 (0.86–1.10)	0.700	43	1.02 (0.94–1.12)	0.599

CSF indicates cerebrospinal fluid; *mRS*, modified Rankin Scale; *NLR*, neutrophil-to-lymphocyte ratio; *OR*, odds ratio; and *WBC*, white blood cells

_*_All ORs are adjusted for severity index and percent IVH clearance

_†_Median cell counts are from ≥3 daily counts

_‡_Median/100

_#_Cell counts are in units of cells per μL

### Sensitivity analysis

Sensitivity analysis was performed considering only those patients with IVH volume ≥20 mL. At day 30, CSF WBC (*P=*0.014), CSF neutrophils (OR=1.01 [95% CI, 1.00–1.03], *P=*0.042), CSF monocytes (*P*=0.028), and CSF lymphocytes (*P*=0.029) were all associated with poor mRS for the saline group (Supplementary Table VI). Serum NLR was associated with worse functional outcome in the alteplase group (*P*=0.044). At day 180, serum lymphocyte count was associated with good functional outcomes in the saline group (*P=*0.002).

### Associations with IVH clearance

When considering all patients as well as the saline and alteplase groups individually, there was generally no significant association between median serum and CSF WBC count, neutrophils, monocytes, or NTL ratio and percent IVH clearance (Supplementary Table VII). Serum lymphocyte count was associated with increased percent IVH clearance only in the alteplase group (*P=*0.015). CSF lymphocyte count was associated with percent IVH clearance both overall (*P=*0.033) and in the saline group (*P=*0.019) independent of stability IVH volume. There was no association between each cell type and parenchymal ICH volumes across treatment groups (data not shown).

### Association with infection

Given the association between serum lymphocyte counts and functional outcomes, we evaluated association between median and maximum lymphocyte counts and systemic infectious complications (Supplementary Table VIII). Patients with ventriculitis were excluded from this analysis. Considering all patients, those with any infection within 7 days after ICH had lower median serum lymphocyte counts than those without any infection (*P*=0.007). In the saline group with infections in first week (*n*=40), there was no significant difference in median serum lymphocyte counts between those with and without infection (*P*=0.096). In the alteplase group, those with infection in the first week (*n*=29) had significantly lower median serum lymphocyte counts compared with those without infection (*P=*0.036). Patients with infection also had lower maximum serum lymphocyte counts compared with those with no signs of infection at 7 days (*P*=0.024). There were no significant associations between median CSF lymphocyte counts and 7-day infections. Similar results were found for 30-day infections. Patients with infection within the first week in the alteplase group, however, had lower maximum CSF lymphocyte counts (*P=0.028)*. Scatter plots of cell counts for lymphocytes in association with the presence of infection within the first week are shown in Supplementary Fig. 2G–I.

## Discussion

This work builds on our previous analysis of CSF WBC measures for the time course of inflammatory response and relevant associations in participants in the CLEAR III trial. The prior analysis found that aseptic CSF inflammation after IVH is primarily dependent on the initial IVH volume and was enhanced by thrombolysis, although without detrimental effect on clinical outcome [8]. We now investigate leukocyte subtypes in both serum and CSF and associations of this inflammatory response with relevant outcomes in patients enrolled in CLEAR III. While studies have reported the importance of serum leukocyte subtypes on ICH outcomes [12, 19], this work represents the first assessment of CSF differential cell counts in the context of IVH. We show early elevations in CSF total WBC and all leukocyte subtypes with earlier and significantly more pronounced elevations in all counts in alteplase-treated patients compared to saline-treated patients. No associations were found between CSF cell counts and outcomes for the full cohort. In saline-treated patients with IVH volume >20 mL, higher CSF cell counts (WBC, neutrophils, monocytes, and lymphocytes) were associated with poor 30-day functional outcomes for all leukocyte subtypes, but not at day 180. Higher serum lymphocyte counts and lower NLR were associated with good functional outcomes in this study. Higher serum lymphocyte counts were also associated with fewer infections in the first week.

Several studies have documented the proinflammatory properties of thrombolytic drugs and in particular the development of sterile inflammation after intraventricular alteplase administration [9, 10]. One study also reported a peak in CSF WBC at about 24 h after intraventricular alteplase administration, associated with a concomitant increase in proinflammatory cytokines [9]. While experimental studies suggest that alteplase exposure to the cortex is neurotoxic and causes damage to the blood brain barrier (BBB) [20], we have not observed an independent effect of CSF pleocytosis on outcomes in alteplase-treated patients. In our previous study, none of the measures of CSF WBC counts were associated with functional outcome or mortality [8]. In the current study, no CSF leukocyte subtypes were associated with outcomes in the alteplase-treated cohort. We observed the peak increase in CSF cell counts one day after first alteplase administration, with subsequent reduction toward saline-treated values despite probable ongoing high CSF alteplase concentrations given the every 8-h dosing regimen and persistence of alteplase in the CSF for 12 h or more [21]. The initial peak in all leukocyte subtypes in alteplase-treated patients could be due to release of hemorrhage-driven leukocytes trapped in the clot and released by alteplase or to pro-inflammatory effects of clot degradation. This leukocyte elevation is dominated by neutrophils which are known to be the first recruited hematogenous cells in ICH. By day 2, a similar spike in all leukocyte populations is seen in the saline-treated group, suggesting a delayed response in the absence of alteplase. These leukocyte elevations in saline treated patients on day 2 especially for monocytes and lymphocytes show significant variability, and none achieve statistical significance compared to alteplase-treated patients.

We report associations between serum lymphocyte count and good functional outcome at 180 days in all patients and in the saline group. Both median and maximum serum lymphocyte counts were associated with lower 7- and 30-day total infections. Serum NLR was associated with worse 180-day functional outcome in all and saline-treated patients, consistent with prior reports [22, 23]. The role of lymphocytes in the injured brain is complex and not clearly defined. T cells form a significant proportion of the leukocytes trafficking to the brain after ICH. Mouse models demonstrate that T cells begin to infiltrate the brain on the first day after ICH and peak around 5 days [24–26] and have been found in the CSF after ICH in humans as early as 6 h [27]. T cells comprise a heterogenous population and have been shown to have both protective and deleterious roles [28]. In ICH, both proinflammatory (γδT cells) and immunosuppressive regular T cells (Tregs) traffic to the area of hemorrhage [29]. γδT cells have been shown to contribute to injury after ischemic stroke mainly due to the production of IL-17 [30]. Tregs may provide benefits against the late tissue damage [31], while in the earlier time frame they have been shown to modulate the activity of invading proinflammatory cells [32]. While our study is not able to distinguish between T cell subtypes, it is possible that the increased serum lymphocytes associated with good functional outcomes are primarily Tregs. The lack of association between good mRS outcomes and CSF lymphocytes may be consistent with the fact that most γδT cells do not enter the brain parenchyma but rather accumulate in the leptomeninges and control trafficking of other inflammatory cells [33]. We found an association between CSF lymphocyte count and percent IVH clearance, which may be due to crosstalk between lymphocytes and the coagulation system. In addition to crosstalk with platelets [34], regulatory T cells producing SPARC (secreted protein acidic and rich in cysteine) are crucial for blood clot resorption [35] and may contribute to reduction of hemorrhage volume.

The lack of association between cell counts and mRS outcomes in the alteplase group may be explained by the ability of alteplase to fundamentally alter the innate immune response. The coagulation system works together with innate immunity in response to infection [36, 37], with procoagulant proteinases such as thrombin increasing endothelial cell junction permeability and inducing the expression of proinflammatory cytokines [37]. Conversely, the anticoagulation system has the opposite effect and can decrease inflammation [38]. Alteplase has been shown to have similar anti-inflammatory effects. Tissue plasminogen activator has been shown to antagonize the effects of lipopolysaccharide on macrophages through its ability to reverse phosphorylation of IκBα [39]. While alteplase increases neutrophil degranulation and MMP-9 production, it also increases levels of protective tissue inhibitor of metalloproteinase [40, 41] Alteplase also suppresses macrophage cytokine production dependent on NMDA-R expression. Sošić et al. demonstrated an increased anti-inflammatory cytokine (TGF-β) compared with proinflammatory cytokine (IL-1β) response in the CSF in patients with primary ICH and IVH treated with recombinant tissue plasminogen activator [42]. Although alteplase increases all CSF leukocyte cell counts, its ability to suppress innate immune cell function and shift toward anti-inflammatory cytokine production may mitigate the effect of increased CSF leukocytes on mRS outcomes. Alternatively, the significant elevations in all leukocyte subtypes induced by alteplase may simply overwhelm the ability to observe specific subtype associations with outcomes. Although our data regarding systemic infections excludes ventriculitis and no changes due to alteplase are seen in systemic cell counts, it is possible that there is some crosstalk between CSF inflammatory cells and the systemic circulation. While of unclear physiological significance, the association between lower maximum CSF lymphocytes and increased risk of systemic infections in the first week may reflect an anti-inflammatory role of alteplase. This would also fit with no associations being seen with 30-day infections given the short half-life of alteplase and completion of the treatment course well ahead of this time point. Alternatively, CSF lymphocyte responses may just reflect those in the peripheral blood.

We confirm the association between higher serum NLR and poor outcomes after ICH [14, 22, 23, 43]. This fits with the notion that lymphocytes (or at least a subset of lymphocytes) may play a protective role while neutrophils may potentiate injury [44]. However, we did not find associations between NLR and outcomes in the CSF. We found that higher lymphocyte count was associated with increased IVH clearance (Supplementary Figure VII). CSF NLR was associated with decreased IVH clearance and increased stability IVH volume; however, this association was lost after controlling for severity index (data not shown). While CSF NLR tended to increase in the alteplase-treated group (Supplementary Figure IV) suggesting that alteplase increased CSF neutrophils counts more than lymphocyte, there were no outcome associations. This may be due to the effects of alteplase on the innate immune system discussed above. We also suspect that the high variability in CSF NLR and high number of zero values may limit our ability to assess the effect of CSF NLR on outcome. Variability in CSF leukocyte subtypes from ventricular catheters may also result from variability in catheter location relative to IVH, CSF drainage rates and composition, and hospital protocol-based differences in sample collection times. The confirmation of serum NLR prognostic utility in large IVH with small ICH is significant, however, as serum NLR may be a reasonable marker of neurotoxicity for clinical protocols involving modulation of neutrophil function as a target for immunotherapy [45].

This study has several limitations. There was site-to-site variability in the collection of both daily CSF results and differential cell counts, resulting in a significant number of patients having missing data. However, the characteristics of the population studied here closely mirrored patient characteristics of the CLEAR III cohort and comparison of cases with and without WBC subtypes found no significant differences. Randomized treatment assignment, a 5-day collection period, and adjudicated outcome assessment unrelated to CSF sampling also reduce potential bias. Since randomization occurred at a median of 51.8 h after ictus in the saline group and 52.2 h in the alteplase group, cell counts in this study do not reflect those immediately after ictus. Daily CSF cultures were not mandated, and the frequent use of antibiotic prophylaxis may have confounded the detection of true infection and its correlation with CSF measures.

## Conclusions

Among patients in the CLEAR III cohort with ICH and IVH, alteplase caused a transient increase in CSF leukocyte counts without an effect on outcomes. Higher serum NLR was associated with worse functional outcomes. Higher serum lymphocyte counts may exert a beneficial effect by mitigating risk of infection. CSF lymphocyte counts may have a positive effect on IVH clearance, while only serum lymphocytes were associated with better functional outcome.

## Supplementary Information

**Additional file 1.** Supplementary Table I Number of data points included in analysis. Supplementary Table II Comparison of demographic variables considering those with ≥3 days CSF differential cell counts versus the entire CLEAR III cohort. Supplementary Table III Day-to-day differences in CSF cell counts including zeros. Supplementary Table IV Day-to-day differences in CSF cell counts excluding zero values. Supplementary Table V Day-to-day differences in CSF cell counts comparing the proportion of non-zero to zero values. Supplementary Table VI Associations between leukocyte subtypes and poor functional outcome (mRS 4–6) in patients with IVH volume ≥20 mL. Supplementary Table VII Associations (β and 95% confidence intervals) between median serum and CSF cell counts for leukocyte subtypes and percent IVH clearance. Supplementary Table VIII Association between serum lymphocyte counts and 30-day infections considering all patients (saline and alteplase groups combined). Supplementary Figure 1 Trends in CSF leukocyte counts considering only those values greater than 0. Supplementary Figure 2 Plots of logistic regression models demonstrating significant associations with outcome and infection.

## Data Availability

The data used and analyzed herein are available from the corresponding author upon reasonable request.

## Abbreviations

ICH: Intracerebral hemorrhage
IVH: Intraventricular hemorrhage
CSF: Cerebrospinal fluid
CLEAR III: Clot Lysis: Evaluating Accelerated Resolution of Intraventricular Hemorrhage
mRS: Modified Rankin Scale
NLR: Neutrophil-to-lymphocyte ratio
EVD: External ventricular drain
WBC: White blood cell
RBC: Red blood cell
IQR: Interquartile range
GCS: Glasgow Coma Scale
CI: Confidence interval
OR: Odds ratio
BBB: Blood-brain barrier
Tregs: Regular T cells
