# Supplementary material for: CSF and serum inflammatory response and association with outcomes in spontaneous intracerebral hemorrhage with intraventricular extension: an analysis of the CLEAR-III Trial
Source: J Neuroinflammation. 2021 Aug 21;18:179. doi: 10.1186/s12974-021-02224-w (PMC8380363; doi:10.1186/s12974-021-02224-w)
Supplement: Supplementary file 1 — Additional file 1. Supplementary Table I Number of data points included in analysis. Supplementary Table II Comparison of demographic variables considering those with ≥3 days CSF differential cell counts versus the entire CLEAR III cohort. Supplementary Table III Day-to-day differences in CSF cell counts including zeros. Supplementary Table IV Day-to-day differences in CSF cell counts excluding zero values. Supplementary Table V Day-to-day differences in CSF cell counts comparing the proportion of non-zero to zero values. Supplementary Table VI Associations between leukocyte subtypes and poor functional outcome (mRS 4–6) in patients with IVH volume ≥20 mL. Supplementary Table VII Associations (β and 95% confidence intervals) between median serum and CSF cell counts for leukocyte subtypes and percent IVH clearance. Supplementary Table VIII Association between serum lymphocyte counts and 30-day infections considering all patients (saline and alteplase groups combined). Supplementary Figure 1 Trends in CSF leukocyte counts considering only those values greater than 0. Supplementary Figure 2 Plots of logistic regression models demonstrating significant associations with outcome and infection. [file 12974_2021_2224_MOESM1_ESM.docx]

**SUPPLEMENTARY MATERIAL**

**Supplemental Tables**

Supplementary Table I. Number of data points included in analysis.

Supplementary Table II. Comparison of demographic variables considering those with ≥3 days CSF differential cell counts versus the entire CLEAR III cohort.

Supplementary Table III. Day-to-day differences in CSF cell counts including zeros.

Supplementary Table IV. Day-to-day differences in CSF cell counts excluding zero values.

Supplementary Table V. Day-to-day differences in CSF cell counts comparing the proportion of non-zero to zero values.

Supplementary Table VI. Associations between leukocyte subtypes and poor functional outcome (mRS 4–6) in patients with IVH volume ≥20 mL.

Supplementary Table VII. Associations (β and 95% confidence intervals) between median serum and CSF cell counts for leukocyte subtypes and percent IVH clearance.

Supplementary Table VIII. Association between serum lymphocyte counts and 30-day infections considering all patients (saline and alteplase groups combined).

**Supplementary Figure**

Supplementary Figure 1. Trends in CSF leukocyte counts considering only those values greater than 0.

Supplementary Figure 2. Plots of logistic regression models demonstrating significant associations with outcome and infection.

**Supplementary Table I:** Number of cell counts available for analysis for days 0-5 post-randomization and number determined to be outliers.

| Cell type | Total cell counts | Outliers | Cell counts analyzed |
| --- | --- | --- | --- |
| WBC | 2539 | 0 | 2539 |
| Neutrophils | 1640 | 0 | 1640 |
| Monocytes | 1640 | 0 | 1640 |
| Lymphocytes | 1640 | 0 | 1640 |
| NTL | 1640 | 0 | 1640 |
| CSF WBC | 1688 | 52 | 1636 |
| CSF Neutrophils | 1512 | 35 | 1477 |
| CSF Monocytes | 1512 | 29 | 1483 |
| CSF Lymphocytes | 1512 | 31 | 1481 |
| CSF NTL | 1512 | 35 | 1477 |

Abbreviations: WBC, white blood cells; NTL, neutrophil-to-lymphocyte ratio; CSF, cerebrospinal fluid

**Supplementary Table II**: Comparison of demographic variables between patients with ≥3 daily differential counts and patients with < 3 daily differential counts.

|  | | Cohort with ≥3 CSF differentials | | Cohort with <3 CSF differentials | |
| --- | --- | --- | --- | --- | --- |
|  | | Alteplase (n=139) | Saline (n=153) | Alteplase (n=108) | Saline (n=100) |
| Demographic variables | | |  |  |  |
| Age (years) | 57 (46-63) | | 58 (51-66) | 60 (51-69) | 60 (54-69) |
| Women | 63(45%) | | 71 (46%) | 43 (40%) | 46 (46%) |
| Race |  | |  |  |  |
| White | 74 (53%) | | 87 (57%) | 70 (65%) | 67 (67%) |
| African  American | 59 (42%) | | 58 (38%) | 35 (32%) | 30 (30%) |
| Native  American | 0 (0%) | | 0 (0%) | 0 (0%) | 0 (0%) |
| Other | 6 (4%) | | 8 (5%) | 3 (2%) | 3 (3%) |
| Ethnicity: Hispanic | 15 (11%) | | 17 (11%) | 13 (12%) | 16 (16%) |
| Baseline Variables | | |  |  |  |
| Tobacco use | 40 (29%) | | 41(27%) | 31 (29%) | 22 (22%) |
| Cocaine | 7 (5%) | | 12 (8%) | 5 (5%) | 7 (7%) |
| Anticoagulation | 15 (11%) | | 20 (13%) | 10 (8%) | 11 (11%) |
| Hypertension | 90 (65%) | | 122 (80%) | 76 (70%) | 81 (81%) |
| Hyperlipidemia | 132 (95%) | | 151(99%) | 105 (97%) | 98 (98%) |
| Antiplatelet use | 28 (20%) | | 38 (25%) | 26 (24%) | 27 (27%) |
| MAP | 96 (88-108) | | 95 (87-105) | 96 (86-107) | 95 (87-104) |
| GCS* | 10 (7-13) | | 10 (7-13) | 8 (6-12) | 9 (7-13) |
| NIHSS | 18 (9-32) | | 19 (10-33) | 17 (9-26) | 18 (11-27) |
| Stability CT |  | |  |  |  |
| IVH volume  (mL) | 21.3 (13.7-35.3) | | 24.8 (16.4, 39.6) | 21.1 (12.3-31.8) | 22.9 (12.9-39.9) |
| ICH volume  (mL) | 8.3 (2.8-15.2) | | 7.7 (2.0-14.2) | 8.8 (3.1-15.5) | 8.2 (2.4-14.4) |
| Index clot location |  | |  |  |  |
| Thalamus | 75 (54%) | | 83 (54%) | 56 (52%) | 55 (55%) |
| Primary IVH | 17 (12%) | | 16 (10%) | 12 (11%) | 9 (9%) |
| Ictus to hospital arrival (h) | 1.5 (0.8-3.3) | | 1.6 (0.8-3.7) | 1.3 (0.8-2.8) | 1.5 (0.9-3.3) |
| Ictus to first CT (h) | 2.3 (1.3-4.7) | | 2.4 (1.4-5.6) | 2.1 (1.4-3.7) | 2.3 (1.4-5.2) |
| Ictus to first EVD (h) | 6.8 (4.5-10.6) | | 7.8 (5.1-11.0) | 6.9 (4.5-11.2) | 7.3 (5.5-12.1) |
| Ictus to stability CT (h) | 43.6 (23.9-58.6) | | 43.8 (28.1-55.5) | 46.7 (30.1-60) | 44.5 (28.0-60.6) |
| Ictus to randomisation (h) | 52.0 (35.9-66.3) | | 52.0 (40.9-66.8) | 54.3 (40.5-68.7) | 54.3 (41.6-68.5) |

**P=0.027*

Abbreviations: MAP, mean arterial pressure; GCS, Glasgow Coma Scale; NIHSS, NIH Stroke Scale; IVH, intraventricular hemorrhage; CT, computed tomography

**Supplementary Table III**: Day-to-day differences in CSF cell counts including zeros.

|  |  |  | Treatment | | |  |  |
| --- | --- | --- | --- | --- | --- | --- | --- |
|  |  | Saline | | Alteplase | | Difference | |
| Leukocyte | Day^b^ | N | Mean (95%CI) ^a^ | N | Mean (95%CI) | Mean (95%CI) | P ^a^ |
| CSF WBC | 0 | 146 | 80.6 (41.0, 121.1) | 155 | 184.1 (83.2, 106.6) | 103.5 (-5.2, 212.3) | 0.062 |
|  | 1 | 150 | 74.8 (43.9, 109.7) | 158 | 521.4 (283.0, 759.9) | **444.6 (203.9, 685.3)** | **<0.001** |
|  | 2 | 158 | 323.6 (162.8, 484.3) | 128 | 183.6 (117.9, 249.2) | -140.0 (-313.6, 33.6) | 0.114 |
|  | 3 | 157 | 102.7 (62.2, 143.3) | 126 | 214.2 (138.7, 289.6) | **111.4 (25.8, 197.1)** | **0.011** |
|  | 4 | 154 | 68.6 (39.0, 98.2) | 114 | 177.0 (110.1, 244.1) | **108.4 (35.2, 181.7)** | **0.004** |
|  | 5 | 117 | 75.2 (43.7, 106.6) | 93 | 135.8 (76.9, 194.7) | 60.6 (-6.2, 127.4) | 0.075 |
| CSF Neutrophils | 0 | 136 | 63.9 (28.7, 99.0) | 142 | 158.6 (67.8, 249.5) | 94.8 ( -2.7, 192.2) | 0.057 |
|  | 1 | 143 | 112.3 (55.6, 168.9) | 141 | 467.2 (230.3, 704.2) | **355.0 (111.3, 598.6)** | **0.004** |
|  | 2 | 138 | 260.7 (128.4, 393.1) | 115 | 148.0 (90.2, 205.7) | -112.7 (-257.1, 31.6) | 0.126 |
|  | 3 | 146 | 131.8 (55.3, 208.4) | 109 | 164.4 (95.7, 233.1) | 32.6 (-70.3, 135.5) | 0.535 |
|  | 4 | 123 | 44.0 (22.5, 65.5) | 107 | 286.4 (144.4, 428.4) | **242.4 (98.8, 386.0)** | **0.001** |
|  | 5 | 100 | 56.1 (30.1, 82.0) | 77 | 99.6 (44.7, 154.6) | 43.6 (-17.2, 104.4) | 0.160 |
| CSF Monocytes | 0 | 136 | 5.5 (2.2, 8.9) | 137 | 7.3 (3.8, 10.8) | 1.7 (-3.1, 6.6) | 0.482 |
|  | 1 | 139 | 7.3 (3.0, 11.7) | 135 | 33.0 (19.7, 46.4) | **25.7 (11.7, 39.8)** | **<0.001** |
|  | 2 | 136 | 47.8 (17.0, 78.5) | 116 | 26.4 (15.3, 37.5) | -21.3 (-54.0, 11.4) | 0.201 |
|  | 3 | 143 | 11.5 (5.6, 17.3) | 109 | 22.2 (14.1, 30.4) | **10.7 (0.7, 20.8)** | **0.036** |
|  | 4 | 121 | 9.6 (4.7, 14.4) | 100 | 25.6 (13.4, 37.7) | **16.0 (2.9, 29.1)** | **0.016** |
|  | 5 | 97 | 9.3 (3.8, 10.8) | 74 | 22.4 (1.0, 32.9) | **13.1 (1.6, 24.6)** | **0.025** |
| CSF Lymphocytes | 0 | 138 | 14.2 (5.4, 22.9) | 136 | 14.1 (6.9, 21.4) | -0.03 (-11.4, 11.4) | 0.995 |
|  | 1 | 139 | 10.5 (5.8, 15.2) | 136 | 32.2 (18.5, 45.9) | **21.7 (7.2, 36.1)** | **0.003** |
|  | 2 | 136 | 57.1 (1.9, 112.3) | 113 | 16.2 (9.6, 22.9) | -40.9 (-96.5, 14.8) | 0.150 |
|  | 3 | 146 | 14.3 (7.8, 20.7) | 108 | 24.2 (14.7, 33.6) | 9.9 (-1.5, 21.3) | 0.089 |
|  | 4 | 119 | 6.8 (3.8, 9.8) | 103 | 16.5 (9.7, 23.3) | **9.7 (2.2, 17.1)** | **0.011** |
|  | 5 | 98 | 10.5 (5.4, 15.6) | 73 | 12.2 (7.4, 17.1) | 1.8 (-5.3, 9.8) | 0.625 |

^a^ Based on general linear model analysis of treatment by day, accounting for within-patient correlations across days

^b^ Days post-randomization

Abbreviations: WBC, white blood cells; CI, confidence interval

**Supplementary Table IV**: Day-to-day differences in CSF cell counts excluding zero values.

|  |  |  | Treatment | | |  |  |
| --- | --- | --- | --- | --- | --- | --- | --- |
|  |  | Saline | | Alteplase | | Difference | |
| Leukocyte | Day^b^ | N | Mean (95%CI) ^a^ | N | Mean (95%CI) | Mean (95%CI) | P ^a^ |
| CSF WBC | 0 | 61 | 214.8 (122.5, 307.1) | 64 | 392.0 (178.9, 605.0) | 177.2 (-55.1, 409.3) | 0.135 |
|  | 1 | 58 | 208.7 (123.1, 294.3) | 69 | 1164.0 (671.3, 1656.8) | **955.3 (455.2, 1455.5)** | **<0.001** |
|  | 2 | 65 | 770.7 (413.4, 1127.8) | 66 | 360.9 (244.2, 477.7) | **-409.7 (-785.5, -33.9)** | **0.033** |
|  | 3 | 62 | 243.3 (152.1, 334.5) | 70 | 405.6 (280.6, 530.7) | **162.4 (7.58, 317.2)** | **0.040** |
|  | 4 | 64 | 137.1 (61.7, 212.4) | 70 | 271.1 (162.2, 380.1) | **134.0 (1.57, 266.5)** | **0.047** |
|  | 5 | 54 | 139.5 (702, 208.8) | 67 | 224.5 (151.0, 298.0) | 85.0 (-16.1, 186.0) | 0.099 |
| CSF Neutrophils | 0 | 56 | 166.6 (88.4, 244.7) | 61 | 339.3 (144.8, 533.8) | 172.7 (-36.9, 382.3) | 0.106 |
|  | 1 | 59 | 288.0 (159.9, 416.2) | 61 | 1034.0 (548.5, 1519.6) | **746.0 (243.9, 1248.2)** | **0.004** |
|  | 2 | 54 | 657.6 (347.4, 967.9) | 61 | 286.4 (199.6, 373.1) | **-371.3 (-693.4, -49.1)** | **0.024** |
|  | 3 | 56 | 319.4 (132.7, 506.1) | 61 | 297.2 (187.0, 407.5) | -22.1 (-238.9, 194.7) | 0.841 |
|  | 4 | 51 | 89.7 (45.8, 133.6) | 66 | 465.3 (238.9, 691.7) | **375.5 (144.9, 606.2)** | **0.001** |
|  | 5 | 48 | 104.6 (58.5, 150.7) | 56 | 157.5 (105.7, 209.3) | 52.9 (-16.4, 122.3) | 0.134 |
| CSF Monocytes | 0 | 41 | 14.7 (6.35, 23.0) | 55 | 17.9 (9.95, 25.9) | 3.28 (-8.25, 14.8) | 0.577 |
|  | 1 | 53 | 19.0 (8.26, 29.8) | 58 | 76.8 (49.4, 104.2) | **57.8 (28.4, 87.3)** | **<0.001** |
|  | 2 | 50 | 129.8 (50.9, 208.8) | 61 | 50.4 (31.1, 69.8) | -79.4 (-160.7, 1.89) | 0.056 |
|  | 3 | 51 | 32.0 (17.0, 46.9) | 58 | 42.4 (28.7, 56.1) | 10.4 (-9.85, 30.7) | 0.314 |
|  | 4 | 48 | 24.0 (12.8, 35.1) | 56 | 48.1 (26.8, 69.4) | **24.2 (0.12, 48.2)** | **0.049** |
|  | 5 | 45 | 20.5 (10.9, 30.0) | 51 | 33.7 (18.8, 48.6) | 13.3 (-4.46, 31.0) | 0.142 |
| CSF Lymphocytes | 0 | 56 | 70.2 (14.7, 125.7) | 52 | 38.8 (16.0, 61.6) | -31.4 (-91.4, 28.6) | 0.305 |
|  | 1 | 53 | 67.3 (12.9, 121.7) | 60 | 68.8 (41.8, 95.9) | 1.51 (-59.3, 62.3) | 0.961 |
|  | 2 | 52 | 107.6 (35.5, 179.8) | 55 | 25.2 (8.98, 41.4) | **-82.4 (-156.4, -8.51)** | **0.029** |
|  | 3 | 55 | 53.0 (-1.62, 107.6) | 59 | 43.1 (26.0, 60.2) | -9.86 (-67.1, 47.4) | 0.736 |
|  | 4 | 42 | 41.1 (-12.8, 94.9) | 57 | 27.3 (11.2, 43.5) | -13.7 (-69.9, 42.5) | 0.632 |
|  | 5 | 45 | 47.0 (-6.28, 100.4) | 49 | 25.2 (12.8, 37.5) | -21.9 (-76.6, 32.8) | 0.433 |
| CSF NLR | 0 | 56 | 2.41 (0.88, 3.94) | 52 | 8.02 (2.69, 13.4) | 10.9 (-0.57, 22.4) | 0.091 |
|  | 1 | 53 | 4.01 (1.56, 6.46) | 60 | 15.7 (6.77, 24.6) | 9.18 (0.75, 17.6) | **1.52e-4** |
|  | 2 | 52 | 5.25 (2.92, 7.58) | 55 | 10.5 (6.22, 14.8) | 5.25 (3.60, 6.90) | **0.0037** |
|  | 3 | 55 | 5.75 (3.45, 8.05) | 59 | 7.84 (2.92, 12.8) | 1.39 (-5.37, 8.15) | 0.152 |
|  | 4 | 42 | 3.11 (1.34, 4.88) | 57 | 16.5 (6.94, 26.1) | 13.7 (2.64, 24.8) | **1.07e-6** |
|  | 5 | 45 | 2.01 (0.22, 3.80) | 49 | 6.64 (2.69, 10.6) | 3.86 (0.66, 7.06) | **0.0046** |

^a^ Based on general linear model analysis of treatment by day, accounting for within-patient correlations across days

^b^ Days post-randomization

Abbreviations: WBC, white blood cells; CI, confidence interval

**Supplementary Table V**: Day-to-day differences in CSF cell counts comparing the proportion of non-zero to zero values.

|  |  |  | Treatment | | |  |  |
| --- | --- | --- | --- | --- | --- | --- | --- |
|  |  | Saline | | Alteplase | | Odds Ratio | |
| Leukocyte | Day^b^ | N | Mean (95%CI) ^a^ | N | Mean (95%CI) | OR (95%CI) | P ^a^ |
| CSF WBC | 0 | 146 | 0.417 (0.337, 0.498) | 155 | 0.414 (0.336, 0.491) | 0.983 (0.548, 1.76) | 0.954 |
|  | 1 | 150 | 0.387 (0.309, 0.464) | 158 | 0.439 (0.363, 0.516) | 1.32 (0.742, 2.33) | 0.347 |
|  | 2 | 158 | 0.410 (0.334, 0.487) | 128 | 0.512 (0.427, 0.597) | 1.69 (0.936, 3.05) | 0.082 |
|  | 3 | 157 | 0.397 (0.320, 0.473) | 126 | 0.552 (0.467, 0.637) | **2.22 (1.22, 4.06)** | **0.009** |
|  | 4 | 154 | 0.416 (0.334, 0.498) | 114 | 0.616 (0.530, 0.701) | **2.81 (149, 5.28)** | **0.001** |
|  | 5 | 117 | 0.460 (0.372, 0.548) | 93 | 0.718 (0.630, 0.806) | **3.99 (1.96, 8.12)** | **<0.001** |
|  |  |  |  |  |  |  |  |
| CSF Neutrophils | 0 | 136 | 0.410 (0.326, 0.493) | 142 | 0.430 (0.349, 0.512) | 1.12 (0.603, 2.07) | 0.726 |
|  | 1 | 143 | 0.413 (0.333, 0.493) | 141 | 0.455 (0.374, 0.535) | 1.24 (0.684, 2.26) | 0.474 |
|  | 2 | 138 | 0.390 (0.309, 0.471) | 115 | 0.532 (0.443, 0.621) | **2.10 (1.11, 3.97)** | **0.022** |
|  | 3 | 146 | 0.384 (0.305, 0.463) | 109 | 0.559 (0.468, 0.650) | **2.50 (1.31, 4.77)** | **0.005** |
|  | 4 | 123 | 0.414 (0.329, 0.500) | 107 | 0.617 (0.529, 0.705) | **2.89 (1.49, 5.59)** | **0.002** |
|  | 5 | 100 | 0.479 (0.385, 0.573) | 77 | 0.729 (0.633, 0.825) | **3.95 (1.80, 8.63)** | **0.001** |
|  |  |  |  |  |  |  |  |
| CSF Monocytes | 0 | 136 | 0.377 (0.295, 0.459) | 137 | 0.405 (0.323, 0.487) | 1.16 (0.610, 2.22) | 0.646 |
|  | 1 | 139 | 0.382 (0.302, 0.462) | 135 | 0.427 (0.346, 0.508) | 1.28 (0.682, 2.40) | 0.444 |
|  | 2 | 136 | 0.364 (0.284, 0.445) | 116 | 0.523 (0.434, 0.612) | **2.37 (1.22, 4.57)** | **0.010** |
|  | 3 | 143 | 0.357 (0.279, 0.435) | 109 | 0.533 (0.441, 0.625) | **2.61 (1.33, 5.10)** | **0.005** |
|  | 4 | 121 | 0.392 (0.307, 0.477) | 100 | 0.561 (0.467, 0.653) | **2.48 (1.24, 4.94)** | **0.010** |
|  | 5 | 97 | 0.470 (0.376, 0.565) | 74 | 0.685 (0.586, 0.785) | **3.28 (1.49, 7.23)** | **0.003** |
|  |  |  |  |  |  |  |  |
| CSF Lymphocytes | 0 | 138 | 0.405 (0.322, 0.488) | 136 | 0.381 (0.299, 0.462) | 0.870 (0.448, 1.69) | 0.680 |
|  | 1 | 139 | 0.381 (0.301, 0.461) | 136 | 0.439 (0.358, 0.520) | 1.38 (0.727, 2.64) | 0.322 |
|  | 2 | 136 | 0.380 (0.300, 0.461) | 113 | 0.489 (0.400, 0.579) | 1.84 (0.937, 3.60) | 0.077 |
|  | 3 | 146 | 0.378 (0.299, 0.456) | 108 | 0.544 (0.453, 0.635) | **2.52 (1.28, 4.98)** | **0.008** |
|  | 4 | 119 | 0.359 (0.275, 0.443) | 103 | 0.556 (0.465, 0.648) | **3.02 (1.48, 6.13)** | **0.002** |
|  | 5 | 98 | 0.463 (0.369, 0.557) | 73 | 0.666 (0.565, 0.767) | **3.13 (1.41, 6.97)** | **0.005** |

^a^ Based on logistic regression analysis of treatment by day considering the proportion of non-zero values in the alteplase group relative to the saline group.

^b^ Days post-randomization

Abbreviations: WBC, white blood cells; CI, confidence interval

**Supplementary Table VI.** Odds ratios (95%CI) between median cell counts^a^ for leukocyte subtypes and poor functional outcome (mRS 4-6) in patients with IVH volume ≥20 mL.

|  |  | All Patients | | | Saline | | |  | Alteplase | |
| --- | --- | --- | --- | --- | --- | --- | --- | --- | --- | --- |
| 30-d | **Cell Type** | **N** | **OR (95%CI)** | ***P-*Value** | **N** | **OR (95%CI)** | ***P*-Value** | **N** | **OR (95%CI)** | ***P*-Value** |
| Serum | WBC | 193 | 0.99 (087, 1.14) | 0.958 | 96 | 1.04 (0.81, 1.33) | 0.756 | 97 | 0.97 (0.84, 1.12) | 0.701 |
|  | Neutrophils* | 126 | 1.00 (0.97, 1.03) | 0.946 | 66 | 1.00 (0.96, 1.03) | 0.950 | 60 | 1.00 (0.96, 1.05) | 0.870 |
|  | Monocytes* | 128 | 0.98 (0.79, 1.22) | 0.889 | 66 | 1.02 (0.79, 1.32) | 0.873 | 62 | 0.88 (0.60, 1.27) | 0.489 |
|  | Lymphocytes* | 128 | 0.94 (0.85, 1.04) | 0.201 | 66 | 1.00 (0.88, 1.12) | 0.945 | 62 | 0.84 (0.70, 1.00) | 0.052 |
|  | NTL | 126 | 1.19 (0.92, 1.54) | 0.190 | 66 | 1.03 (0.83, 1.28) | 0.801 | 60 | 1.90 (1.02, 3.55) | **0.044** |
| CSF | WBC* | 80 | 1.30 (0.82, 2.06) | 0.266 | 37 | 9.11 (1.56, 53.2) | **0.014** | 43 | 1.12 (0.76, 1.67) | 0.564 |
|  | Neutrophils* | 74 | 1.30 (0.73, 2.31) | 0.377 | 33 | 3.67 (1.01, 13.4) | **0.049** | 41 | 1.12 (0.76, 1.64) | 0.573 |
|  | Monocytes | 65 | 1.01 (0.95, 1.07) | 0.713 | 31 | 1.20 (1.01, 1.44) | **0.042** | 34 | 1.00 (0.96, 1.04) | 0.950 |
|  | Lymphocytes | 67 | 1.14 (0.95, 1.37) | 0.156 | 30 | 1.72 (1.06, 2.80) | **0.029** | 37 | 1.05 (0.93, 1.19) | 0.427 |
|  | NTL | 46 | 0.99 (0.88, 1.11) | 0.824 | 22 | 0.82 (0.63, 1.06) | 0.127 | 24 | 1.03 (0.90, 1.19) | 0.662 |
|  |  |  |  | |  |  | |  |  | |
| 180-d | **Cell Type** | **N** | **OR (95%CI)** | ***P-*Value** | **N** | **OR (95%CI)** | ***P*-Value** | **N** | **OR (95%CI)** | ***P*-Value** |
| Serum | WBC | 154 | 0.97 (0.88, 1.07) | 0.518 | 71 | 0.92 (0.70, 1.22) | 0.570 | 83 | 0.97 (0.88, 1.08) | 0.613 |
|  | Neutrophils* | 102 | 0.99 (0.97, 1.02) | 0.552 | 49 | 0.97 (0.93, 1.01) | 0.107 | 53 | 1.01 (0.98, 1.04) | 0.592 |
|  | Monocytes* | 102 | 1.02 (0.84, 1.25) | 0.815 | 49 | 1.02 (0.78, 1.33) | 0.870 | 53 | 1.00 (0.70, 1.42) | 0.998 |
|  | Lymphocytes* | 103 | 0.92 (0.83, 1.03) | 0.153 | 49 | 0.76 (0.64, 0.90) | **0.002** | 54 | 1.12 (0.94, 1.32) | 0.194 |
|  | NTL | 102 | 1.09 (0.91, 1.31) | 0.339 | 49 | 1.28 (0.96, 1.69) | 0.087 | 53 | 0.95 (0.75, 1.20) | 0.648 |
| CSF | WBC* | 59 | 1.06 (0.95, 1.18) | 0.299 | 24 | 0.95 (0.62, 1.46) | 0.825 | 35 | 1.08 (0.90, 1.30) | 0.391 |
|  | Neutrophils* | 56 | 1.25 (0.96, 1.63) | 0.095 | 22 | 2.51 (0.31, 20.2) | 0.387 | 34 | 1.18 (0.96, 1.45) | 0.105 |
|  | Monocytes | 47 | 0.99 (0.96, 1.03) | 0.648 | 20 | 0.99 (0.96, 1.02) | 0.549 | 27 | 1.00 (0.97, 1.04) | 0.819 |
|  | Lymphocytes | 50 | 1.01 (0.98, 1.04) | 0.623 | 20 | 1.01 (0.952, 1.07) | 0.730 | 30 | 1.01 (0.98, 1.04) | 0.621 |
|  | NTL | 34 | 0.98 (0.90, 1.08) | 0.736 | 15 | 0.94 (0.78, 1.13) | 0.507 | 19 | 1.04 (0.91, 1.19) | 0.593 |

^a^ Medians with >= 3 differential measurements per patient

^b^ Odds ratio (95% CI) and p-value are based on logistic regression analyses with robust standard errors.

*Median cell counts/100

Abbreviations: WBC, white blood cells; NTL, neutrophil-to-lymphocyte ratio; OR, odds ratio; CI, confidence interval

**Supplementary Table VII. Associations (β and 95% confidence intervals) between median serum and CSF cell counts for leukocyte subtypes and percent IVH clearance.**

|  |  | **All Patients** | | | **Saline** | | | **Alteplase** | | |
| --- | --- | --- | --- | --- | --- | --- | --- | --- | --- | --- |
|  | **Cell Type*** | **N** | **β^†^ (95% CI)** | ***P*** | **N** | **β (95% CI)** | ***P*** | **N** | **β (95% CI)** | ***P*** |
| **Serum** | WBC | 424 | -0.36 (-1.62, 0.90) | 0.573 | 215 | -0.23 (-1.44, 0.98) | 0.705 | 209 | -0.46 (-2.39, 1.48) | 0.644 |
|  | Neutrophils^‡^ | 275 | -0.14 (-0.35, 0.06) | 0.177 | 149 | -0.04 (-0.24, 0.16) | 0.695 | 126 | -0.24 (-0.59, 0.12) | 0.192 |
|  | Monocytes^‡^ | 277 | -0.59 (-1.69, 0.50) | 0.285 | 149 | 0.08 (-1.37, 1.53) | 0.913 | 128 | -1.23 (-2.68, 0.23) | 0.097 |
|  | Lymphocytes^‡^ | 278 | 0.30 (-0.44, 1.04) | 0.423 | 149 | -0.45 (-1.38, 0.48) | 0.342 | 129 | 1.29 (0.25, 2.33) | **0.015** |
|  | NTL | 275 | -1.25 (-2.60, 0.10) | 0.070 | 149 | -0.29 (-1.49, 0.92) | 0.639 | 126 | -2.47 (-5.23, 0.28) | 0.078 |
| **CSF** | WBC^‡^ | 156 | 0.07 (-0.26, 0.40) | 0.687 | 72 | 0.36 (-2.00, 2.72) | 0.762 | 84 | 0.03 (-0.28, 0.35) | 0.832 |
|  | Neutrophils^‡^ | 146 | 0.16 (-0.44, 0.75) | 0.603 | 65 | 0.57 (-2.65, 3.79) | 0.725 | 81 | 0.10 (-0.48, 0.68) | 0.734 |
|  | Monocytes | 131 | 0.04 (-0.10, 0.18) | 0.558 | 60 | 0.08 (-0.15, 0.31) | 0.472 | 71 | 0.02 (-0.14, 0.19) | 0.777 |
|  | Lymphocytes | 130 | 0.09 (0.01, 0.16) | **0.033** | 57 | 0.08 (0.01, 0.14) | **0.019** | 73 | 0.11 (-0.08, 0.31) | 0.243 |
|  | NTL | 96 | -0.06 (-0.79, 0.67) | 0.872 | 43 | -0.03 (-0.09, 0.09) | 0.947 | 53 | -0.05 (-1.07, 0.10) | 0.930 |

CSF indicates cerebrospinal fluid; NTL, neutrophil-to-lymphocyte ratio; and WBC, white blood cells.
* Median cell counts are from ≥3 daily counts.
^†^ Each model was adjusted for severity index.
^‡^ Median/100.

**Supplementary Table VIII.** Association between serum and CSF lymphocyte counts and 7- and 30-day infections.

|  |  | 7d Infection (No Ventriculitis days 0-6) | | | | | 30d Infection | | | | |
| --- | --- | --- | --- | --- | --- | --- | --- | --- | --- | --- | --- |
|  |  | No Infection | | Infection | | P^b^ | No Infection | | Infection | | P |
| Lymphocyte Count | Group | N^a^ | Mean (SD) | N | Mean (SD) |  | N | Mean (SD) | N | Mean (SD) |  |
| Median Serum | All | 210 | 1433 (528) | 69 | 1282 (536) | **0.007** | 142 | 1455 (540) | 140 | 1330 (533) | **0.014** |
|  | Saline | 110 | 1412 (534) | 40 | 1309 (611) | 9.096 | 73 | 1416 (530) | 78 | 1348 (579 ) | 0.210 |
|  | Alteplase | 100 | 1455 (523) | 29 | 1246 (503) | **0.036** | 69 | 1496 (551) | 62 | 1307 (473) | **0.028** |
|  |  |  |  |  |  |  |  |  |  |  |  |
| Median CSF | All | 205 | 9.60 (28.6) | 67 | 5.47 (11.25) | 0.356 | 143 | 10.29 (32.9) | 142 | 16.63 (61.1) | 0.533 |
|  | Saline | 107 | 7.09 (32.3) | 38 | 6.51 (13.4) | 0.749 | 77 | 7.78 (34.5) | 74 | 17.77 (79.5) | 0.399 |
|  | Alteplase | 98 | 12.35 (26.7) | 29 | 4.11 (7.6) | 0.097 | 66 | 13.21 (31.0) | 68 | 15.39 (31.4) | 0.938 |
|  |  |  |  |  |  |  |  |  |  |  |  |
| Maximum Serum | All | 210 | 1881 (729) | 69 | 1701 (729) | **0.024** | 142 | 1910 (721) | 140 | 1755 (734) | **0.023** |
|  | Saline | 110 | 1864 (792) | 40 | 1730 (793) | 0.138 | 73 | 1868 (756) | 78 | 1780 (827) | 0.188 |
|  | Alteplase | 100 | 1899 (657) | 29 | 1661 (643) | 0.101 | 69 | 1954 (686) | 62 | 1723 (602) | 0.069 |
|  |  |  |  |  |  |  |  |  |  |  |  |
| Maximum CSF | All | 205 | 76.78 (273.4) | 67 | 44.58 (79.4) | 0.406 | 143 | 470.3 (4445) | 142 | 140.0 (348) | 0.892 |
|  | Saline | 107 | 79.90 (366.8) | 38 | 55.89 (97.5) | 0.364 | 77 | 796.1 (6055) | 74 | 144.4 (358) | 0.200 |
|  | Alteplase | 98 | 73.36 (100.7) | 29 | 29.76 (43.5) | **0.028** | 66 | 90.2 (145) | 68 | 135.1 (341) | 0.286 |

| ^a^ N's include patients with counts on at least 3 days |
| --- |
| ^b^ Based on Mann-Whitney test to compare distributions of two groups. Bold values have P <0.05 |
| Abbreviations: CSF, Cerebrospinal Fluid |





Supplementary Figure 1. Trends in CSF leukocyte counts including only those values greater than 0. Mean cell counts with standard deviations are shown for both alteplase and saline groups. Day 0 represents the day of randomization and first day of treatment with subsequent values over the next 5 days. *P* values are shown for each cell type and represent differences in overall trends between the two groups. Significance (saline vs alteplase): **P*≤0.001, ***P*<0.005, ^†^*P*<0.05.


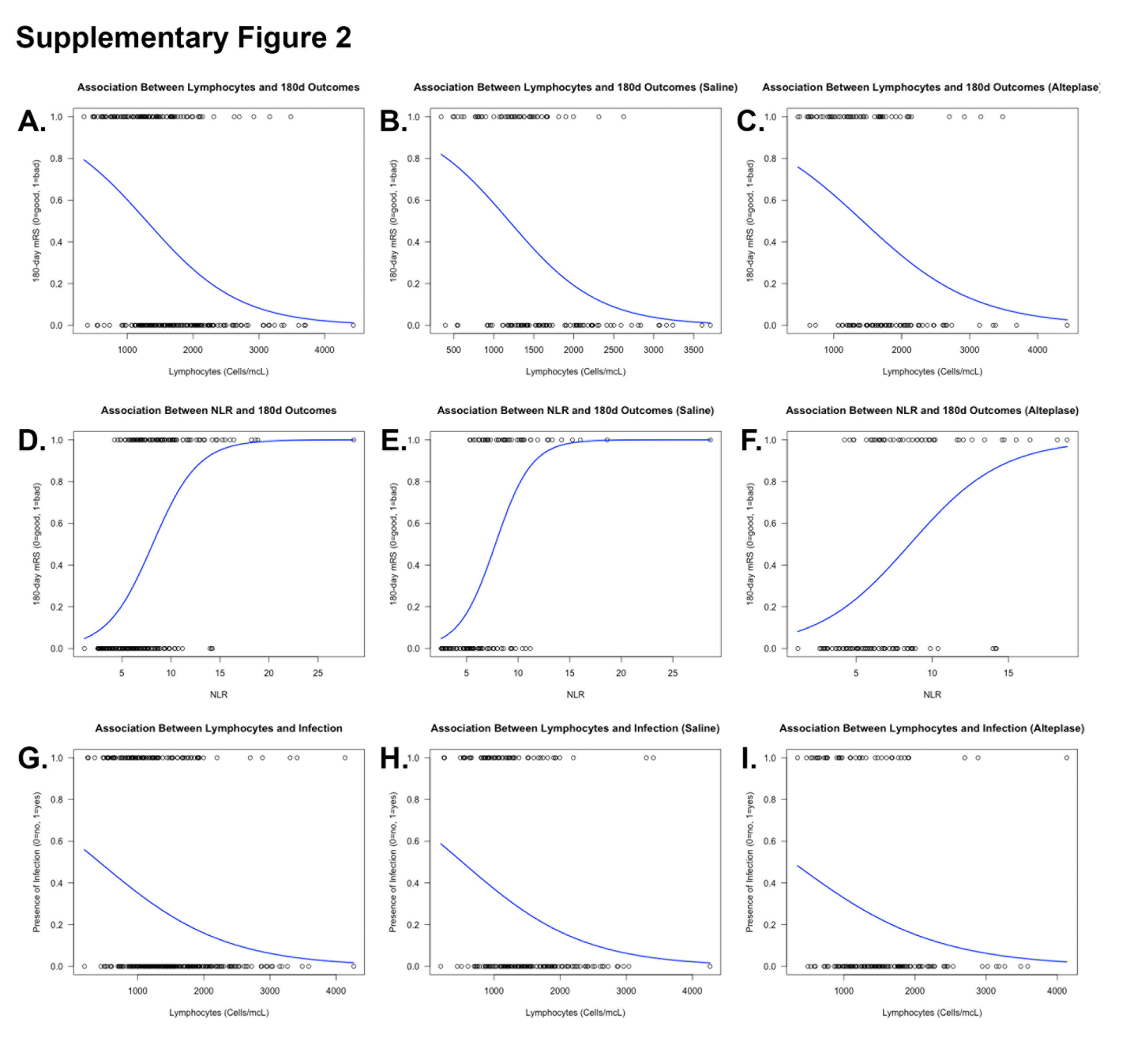


Supplementary Figure 2. Plots of logistic regression models demonstrating significant associations between serum cell counts and outcome and infection. For panels A-F, 0 corresponds to a good functional outcome at 180-days, while 1 corresponds to a poor functional outcome at 180-days. For panels G-I, 0 corresponds to absence of infection within the first week, while 1 corresponds to presence of infection. Outcomes associations are shown for lymphocytes in all subjects (A), lymphocytes in the saline group (B), lymphocytes in the alteplase group (C), neutrophil-to-lymphocyte ratio (NLR) in all subjects (D), NLR in the saline group (E), and NLR in the alteplase group (F). Associations between infection and lymphocytes are shown in all subjects (G), the saline group (H), and the alteplase group (I).
